# Supplementary material for: Genomic Characteristics and Functional Analysis of Brucella sp. Strain WY7 Isolated from Antarctic Krill
Source: Microorganisms. 2023 Sep 11;11(9):2281. doi: 10.3390/microorganisms11092281 (PMC10536100; doi:10.3390/microorganisms11092281)
Supplement: Supplementary file 1 [file microorganisms-11-02281-s001.zip › microorganisms-2529844-supplementary/Supplementary-Figure Legends-V.pdf]

Supplementary Figure and Legends

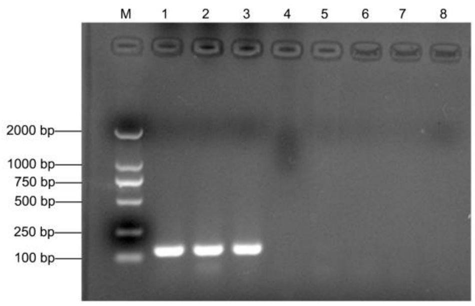

Figure S1. Detection of specific primers for *Brucella* sp. WY7 gene1983. M: DS2000 Marker; 1: *Brucella* sp. WY7 DNA; 2: WY7 bacterial fluid 1; 3: WY7 bacterial fluid 2; 4: *Brucella anthropi* MCCC 1K05807; 5: *Brucella anthropi* ATCC 49188; 6: *Escherichia coli* ATCC 25922; 7: *Staphylococcus aureus* ATCC 25923; 8: H_2O. The length of the target fragment is 154 bp. Gene1983 is defined as the gene participating in any process that activates or increases the frequency, rate or extent of cellular DNA-templated transcription.

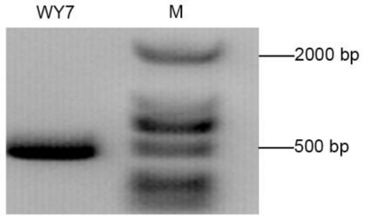

Figure S2. In situ identification of specific primers from the strain WY7. M: marker DS2000, WY7: *Brucella* sp. WY7.
