# Supplementary figures and images for: Genomic Characteristics and Functional Analysis of Brucella sp. Strain WY7 Isolated from Antarctic Krill
Source: Microorganisms. 2023 Sep 11;11(9):2281. doi: 10.3390/microorganisms11092281 (PMC10536100; doi:10.3390/microorganisms11092281)

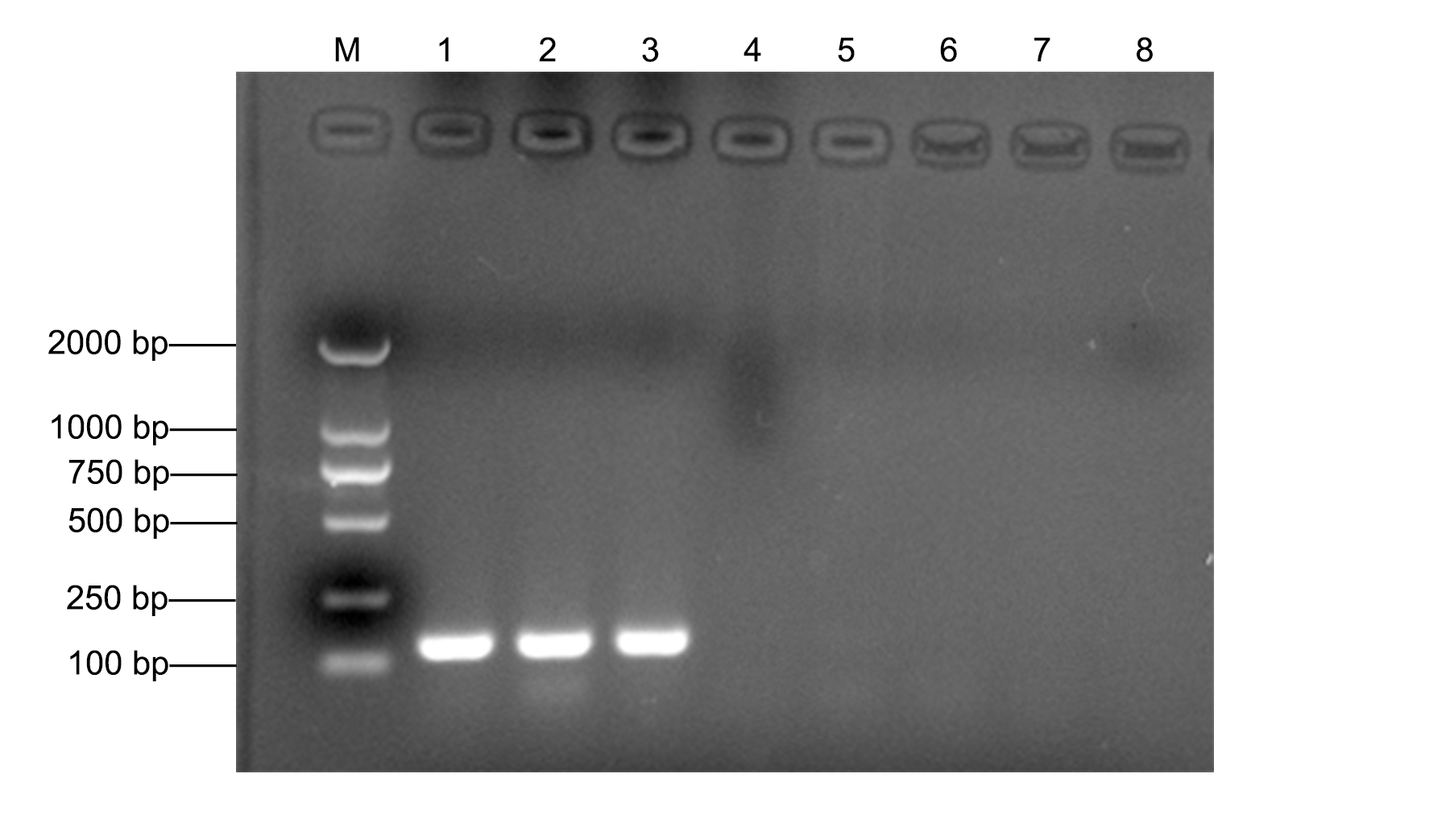

Supplement: Supplementary file 1 [file microorganisms-11-02281-s001.zip › microorganisms-2529844-supplementary/Figure S1.tif]

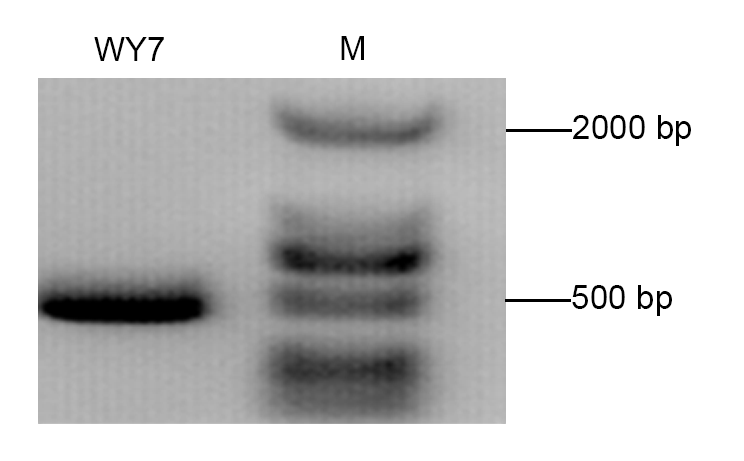

Supplement: Supplementary file 1 [file microorganisms-11-02281-s001.zip › microorganisms-2529844-supplementary/Figure S2.tif]
